# The Misuse of Hospitals;—Consultants and General Practitioners

**Published:** 1895-07-20

**Authors:** 


### The Hospital

An Institutional Journal of

The Medical Sciences and Hospital Administration, with

AN EXTRA NURSING SUPPLEMENT.

Vol. XVIII.—No. 460.] [July 20, 1895.

### The Misuse of Hospitals;—Consultants and General Practitioners.

Last week we pointed out the inordinate growth in the number of patients receiving relief at the metropolitan hospitals as compared with the increase in the population during the last twenty years. We showed that the system of admitting patients to the metropolitan hospitals required reform and amendment; that the present system is bringing the general practitioners more and more into antagonism with the honorary medical officers of the hospitals; and that a series of conferences between these two sections of the medical profession is earnestly called for.

Dealing first with the conference, the cardinal factor in the situation is that the general practitioners have the remedy in their own hands. The present hospital system tends to cripple their means of livelihood, to place them in antagonism with the consultants and the voluntary hospitals, and to make them discontented, and therefore less useful members of the community. If it were not for the existence of hospitals it would be impossible to train men for the medical profession. The great medical schools could not exist without the hospitals, and the general practitioners who have issued from these schools have a paramount claim for consideration upon the honorary medical staff of the great hospitals where they received their training. But further and even stronger are the pecuniary ties which bind the consultants to the general practitioners of medicine. The consultant depends very largely for his income upon the patients which are sent to him in consultation by the general practitioners who were educated at the school where he is a teacher and honorary medical officer. We therefore desire that the publication of our Special Supplement, combined with the instructive and accurate figures and facts to be found in Burdett's "Hospital and Charities Annual, 1895," may be promptly considered by the members of the medical profession. We hope that the more active general practitioners will associate themselves together to form a small executive committee in connection with each of the great medical schools of which they were students, that they will go into the questions thus raised, will prepare a memorial to the honorary medical staff and teachers embodying the facts, and that they will then submit it to a general meeting of all the post-graduates of their *alma mater* for approval and criticism. When the memorial is thus perfected a deputation of the most representative and influential of the post-graduates of the particular school should place themselves in communication with the medical authorities of the hospital and invite a conference on the questions at issue.

What, then, are the questions which may properly be submitted for the decision and elucidation of such conferences? Undoubtedly, the first and paramount question is payment by the hospitals for all medical service rendered to the patients who flock to them. It was never contemplated, when the present system of voluntary hospitals first arose, that one in every two of the population would ultimately find their way to these institutions for medical treatment. In the early days hospitals were unpopular, and no one would enter them unless compelled to do so from dire necessity or accident. As the system of management has been improved and perfected, people have learnt to have the utmost confidence in the hospitals, and not one but all classes of society now regard a voluntary hospital as the safest and best place of treatment for a sick person when seriously ill. It follows that as the voluntary hospitals have been established upon the broad principle of free medical relief to all or nearly all patients, a large and increasing number of people have every year obtained free medical relief to which they were not entitled by their circumstances, or even at all. The hospital authorities twenty years ago, when the home hospital movement began, contended that it was contrary to their constitution and objects to admit patients for treatment on payment of the whole or even a part of the cost which it entailed. The success of the first Home Hospital, Fitzroy House, Fitzroy Square, has educated hospital and public opinion, and at the present time the general hospitals throughout the country have gradually come to recognise the soundness of the principle of paying wards, and they derive on an average nearly 3 per cent. of their ordinary income from patients' payments at the present time. It is noticeable, however, that the general hospitals of London receive less than 2 per cent. of their income, although the Scotch general hospitals receive 5½ per cent., and the Irish 8 per cent. from this source. This hesitancy on the part of the metropolitan general hospitals to adopt the pay system is due, no doubt, to the conservatism of their management and the absence of any proved necessity to introduce a new system in order to provide adequate funds. In the case of cottage hospitals, of which there are now many hundreds in England, the managers have recognised from the first the importance of permitting every patient to pay something, however small, towards his maintenance. Although the patients treated in cottage hospitals come, if possible, from a poorer class than those to be found in town hospital wards, the patients' payments amount to at least 12 per cent. of the income of these institutions. During the last thirty years special hospitals have increased and multiplied. The managers had no endowments to fall back upon like the general hospitals, and as they had to wholly depend upon popular support for their maintenance, they speedily adopted a system of patients' payments, which yields in London at the present time about 11½ per cent., and in the provinces about 17⅔ per cent. of the total income of these special hospitals.

On financial grounds there is therefore the strongest argument in favour of the institution of paying wards in connection with the general hospitals. The public, that is, all classes of people in England to-day, are prepared to welcome the introduction of the paying ward, and to pay for their treatment there when ill such fees as the authorities may consider adequate and proper. The public declare, and we think rightly, that as their individual interests demand that they shall have the best medical treatment when they are seriously ill, they intend to go to the hospitals where they are sure to get it, and if the hospitals will not permit them to pay for it, then they will pocket their feelings and accept free relief. This is the kernel of the present position as represented by the sentiments of the great mass of the people. They recognise—and we have had two instances brought under our notice in the last fortnight which support this view—that when a patient who is able to pay, to a certain extent, for medical treatment at home is dangerously ill, especially from some surgical malady, the treatment which he can obtain for payment at home is not equal to the treatment which the general hospitals can offer him. Self-preservation is the first law of nature, and the natural man and the natural woman, too, in these days of education realises the risks of remaining at home, and declines to take them. Here we have the true explanation of the ever-increasing demand upon the resources of the voluntary hospitals, and that demand is not met as it ought to be met in the best interests of the patients and the profession, by providing pay wards and remuneration to the medical attendant, mainly because hospital medical officers are at present honorary and unpaid. Once pay the medical staff of all the hospitals, and you will destroy for ever not only hospital abuse, but hospital extravagance too.

